# Detailed insights into pan‐European population structure and inbreeding in wild and hatchery Pacific oysters (*Crassostrea gigas*) revealed by genome‐wide SNP data

**DOI:** 10.1111/eva.12736

**Published:** 2018-12-31

**Authors:** David L. J. Vendrami, Ross D. Houston, Karim Gharbi, Luca Telesca, Alejandro P. Gutierrez, Helen Gurney‐Smith, Natsuki Hasegawa, Pierre Boudry, Joseph I. Hoffman

**Affiliations:** ^1^ Department of Animal Behavior Bielefeld University Bielefeld Germany; ^2^ The Roslin Institute and Royal (Dick) School of Veterinary Studies University of Edinburgh Midlothian UK; ^3^ Edinburgh Genomics, Ashworth Laboratories University of Edinburgh Edinburgh UK; ^4^ Department of Earth Sciences University of Cambridge Cambridge UK; ^5^ British Antarctic Survey, High Cross Cambridge UK; ^6^ Department of Fisheries and Aquaculture Vancouver Island University Nanaimo British Columbia Canada; ^7^ National Research Institute of Aquaculture Japan Fisheries Research Agency Minami‐Ise Japan; ^8^ Ifremer Laboratoire des Sciences de l’Environnement Marin (UBO/CNRS/IRD/Ifremer) Plouzané France

**Keywords:** aquaculture, *Crassostrea gigas*, genetic structure, high‐density genotyping array, inbreeding, Pacific oyster, restriction site‐associated DNA (RAD) sequencing, single nucleotide polymorphism (SNP)

## Abstract

Cultivated bivalves are important not only because of their economic value, but also due to their impacts on natural ecosystems. The Pacific oyster (*Crassostrea gigas*) is the world's most heavily cultivated shellfish species and has been introduced to all continents except Antarctica for aquaculture. We therefore used a medium‐density single nucleotide polymorphism (SNP) array to investigate the genetic structure of this species in Europe, where it was introduced during the 1960s and has since become a prolific invader of coastal ecosystems across the continent. We analyzed 21,499 polymorphic SNPs in 232 individuals from 23 localities spanning a latitudinal cline from Portugal to Norway and including the source populations of Japan and Canada. We confirmed the results of previous studies by finding clear support for a southern and a northern group, with the former being indistinguishable from the source populations indicating the absence of a pronounced founder effect. We furthermore conducted a large‐scale comparison of oysters sampled from the wild and from hatcheries to reveal substantial genetic differences including significantly higher levels of inbreeding in some but not all of the sampled hatchery cohorts. These findings were confirmed by a smaller but representative SNP dataset generated using restriction site‐associated DNA sequencing. We therefore conclude that genomic approaches can generate increasingly detailed insights into the genetics of wild and hatchery produced Pacific oysters.

## 1 INTRODUCTION

Oysters are among the most economically important aquaculture species, with worldwide annual production exceeding 600,000 tonnes (FAO, http://www.fao.org). In particular, the Pacific cupped oyster (*Crassostrea gigas*), which is native to the Pacific coast of eastern Asia, was introduced into many countries worldwide for commercial cultivation. Starting in the 1960s, *C. gigas* was introduced into Europe to support oyster farming after severe declines of the two previously cultivated oyster species–the Portuguese oyster (*C. angulata*) and the flat oyster (*Ostrea eduli*s, Grizel & Héral, 1991, Nehring, 1999, Wolff & Reise, 2002). Large quantities of seed as well as adult oysters were brought to France and the Netherlands from the Miyagi prefecture in Japan and from British Columbia in Canada, where *C. gigas* was also introduced from Japan in the 1920s (Quayle, 1988) and became quickly established in the wild. Concurrently, several small importations of less than a hundred individuals at a time also took place into the United Kingdom for hatchery propagation (Walne & Helm, 1979).

Subsequently, Pacific oysters produced in UK hatcheries were farmed in the German Wadden Sea (Reise, 1998) as well as in Denmark (Nehring, 2006), while oysters produced in French farms were transferred to various locations in the Mediterranean Sea (Grizel & Héral, 1991; Šegvić‐Bubić et al., 2016) including southern Portugal, where hybridization with *C. angulata* is known to occur (Batista, Fonseca, Ruano, & Boudry, 2017; Huvet, Fabioux, McCombie, Lapegue, & Boudry, 2004). More recently, *C. gigas* also reached the southern coasts of Sweden and Norway (Troost, 2010), where it arrived as a consequence of both natural dispersal from Denmark and human‐mediated translocation from British hatcheries (d'Auriac et al., 2017). Consequently, Pacific oysters have become widespread across the Atlantic and Mediterranean coasts of Europe, where they are responsible for major changes to coastal ecosystems (Troost, 2010) and are considered an invasive species (Goulletquer, Bachelet, Sauriau, & Noel, 2002).

Several studies have used genetic markers such as mitochondrial DNA and microsatellites to investigate the population structure of the Pacific oyster in Europe, mainly with a view toward understanding the history of invasion (d'Auriac et al., 2017; Faust et al., 2017; Lallias et al., 2015; Meistertzheim, Arnaud‐Haond, Boudry, & Thébault, 2013; Rohfritsch et al., 2013) as well as interrelationships between wild populations and hatcheries (Kochmann, Carlsson, Crowe, & Mariani, 2012; Lallias et al., 2015; Moehler, Wegner, Reise, & Jacobsen, 2011). Many of these studies uncovered evidence for two distinct genetic clusters: one in southern Europe (subsequently referred to as the “southern group”) that includes populations from the Mediterranean, Spain, France, the Netherlands, and the south‐western coast of England, and one in northern Europe (subsequently referred to as the “northern group”) that consists of the remaining British, German, and Scandinavian populations (Lallias et al., 2015; Meistertzheim et al., 2013; Moehler et al., 2011; Rohfritsch et al., 2013). Furthermore, no genetic differences were found between the southern group and the source populations of Japan and British Columbia, suggesting that the original mass introduction may not have resulted in a founder effect (Rohfritsch et al., 2013). Additionally, the northern group was found to have lower genetic diversity, suggesting that it probably arose locally in Europe and more specifically in the UK as a consequence of repeated small introduction events that may have acted as bottlenecks due to hatchery propagation followed by genetic drift (Faust et al., 2017; Lallias et al., 2015).

Although previous studies have provided important insights into the population structure of Pacific oysters in Europe, many focused on local scales and, even though Rohfritsch et al. (2013), Lallias et al. (2015), and Faust et al. (2017) sampled extensively along the western Atlantic seaboard, there is still a need for more comprehensive studies encompassing the full latitudinal range of the species in Europe and including hatcheries from both Britain and France. Furthermore, classical approaches such as mitochondrial sequencing and microsatellite genotyping have limited power to detect population structure, especially over fine geographic scales where genetic differences may be too subtle to be captured with a handful of markers (Vendrami et al., 2017). By contrast, new genomic approaches capable of genotyping tens of thousands of single nucleotide polymorphisms (SNPs) have been proven to have far greater power to resolve genetic differences among populations (Morin, Luikart, & Wayne, 2004; Rašić, Filipović, Weeks, & Hoffmann, 2014) and therefore allow more in‐depth studies of population genetic structure.

One of the most commonly used approaches for generating large SNP datasets for nonmodel organisms is to use genotyping by sequencing methods such as restriction site‐associated DNA (RAD) sequencing (Baird et al., 2008), which allows concurrent SNP identification and genotyping via high‐throughput sequencing of flanking regions of restriction enzyme digestion sites dispersed throughout the genome. These methods have democratized the study of population genomics but are not without their disadvantages (da Fonseca et al., 2016) such as the need for extensive bioinformatic processing, high rates of missing data, and the issue of uncertainty in genotype calling, which can affect downstream analyses (Shafer et al., 2017). A convenient alternative where available is therefore to use a medium‐ or high‐density SNP array, in which the probe sequences of many tens or hundreds of thousands of SNPs are “printed” onto a slide against which the genomic DNA is hybridized. SNP arrays typically generate very high‐quality data with relatively few missing genotypes, but they also have some downsides. Arguably, the most important of these is ascertainment bias, which occurs when not all of the genetic diversity present in a population can be captured by the array due to the use of a limited pool of individuals in the original SNP discovery phase (Lachance & Tishkoff, 2013).

Another drawback of small panels of nuclear markers like microsatellites is that their sampling variance is usually too large to accurately quantify variation in inbreeding (Balloux, Amos, & Coulson, 2004). This may be relevant to aquaculture because moderate to high levels of inbreeding have been shown to have detrimental effects on a variety of commercially important traits, such as harvest body size and larval growth, in several species, including turbot (Lyu, Wu, Hu, & Wang, 2018), Pacific white shrimp (Moss, Arce, Otoshi, Doyle, & Moss, 2007), coho salmon (Gallardo, Garcıa, Lhorente, & Neira, 2004), and Pacific abalone (Deng, Liu, Zhang, & Guo, 2005) as well as in flat oysters (Lallias, Boudry, Lapegue, King, & Beaumont, 2010) and Pacific oysters (Evans, Matson, Brake, & Langdon, 2004; Launey & Hedgecock, 2001; Plough & Hedgecock, 2011). Hence, inbreeding depression could conceivably be a problem if aquaculture practices, such as the use of restricted numbers of parents as broodstock and/or the crossing of related individuals, lead to increased inbreeding in cultured populations (Norris, Bradley, & Cunningham, 1999; Taris, Batista, & Boudry, 2007).

Given the limited power of microsatellites to quantify inbreeding, the method of choice until recently has been to derive individual inbreeding coefficients (*f*) from deep pedigrees (Pemberton, 2008). However, pedigrees can be costly and time‐consuming to construct and may also be unworkable for many aquaculture species due to their high fecundity and broadcast spawning life‐histories. Fortunately, recent simulation (Kardos, Luikart, & Allendorf, 2015; Wang, 2016) and empirical (Hoffman et al., 2014; Huisman, Kruuk, Ellis, Clutton‐Brock, & Pemberton, 2016) studies suggest that inbreeding can now be directly and accurately quantified from genomic data, with around ten thousand or more SNPs being preferable under most circumstances even to a high‐quality pedigree. Consequently, the increasing availability of SNP arrays for non‐model species provides an exciting new opportunity to elucidate how different aquaculture practices influence inbreeding, as well as to identify suitable sources of individuals for use as broodstock to establish effective management and breeding protocols.

Recently, Gutierrez et al. (2017) developed a medium‐density combined species SNP array for Pacific and flat oysters (*Ostrea edulis*). Whole genome sequencing of pooled genomic samples from eight European *C. gigas* populations led to the discovery of 1.2 million putative SNPs, of which 40,625 were printed on the array and 27,697 were validated as being polymorphic and of high quality. This array represents an important resource for selective breeding programs as well as more generally for population genetic studies of oysters. We therefore used it to investigate population genetic structure and inbreeding in *C. gigas* sampled from wild European populations and hatcheries. Specifically, we genotyped 192 individuals from 13 populations spanning a European latitudinal cline from Portugal in the south to Norway in the north. We then incorporated existing data from Gutierrez et al. (2017) to generate a combined dataset of 273 individuals sampled from 23 populations, of which just over half were wild. Our results may be useful for designing exchanges among hatcheries, identifying potential sources of broodstock, and for the elaboration of other effective management strategies aimed at minimizing inbreeding within hatchery propagated Pacific oysters. Consequently, we believe this study provides important information for breeding programs as well as a baseline for future studies.

## 2 MATERIALS AND METHODS

### 2.1 Sample collection and DNA extraction

**Figure 1:**
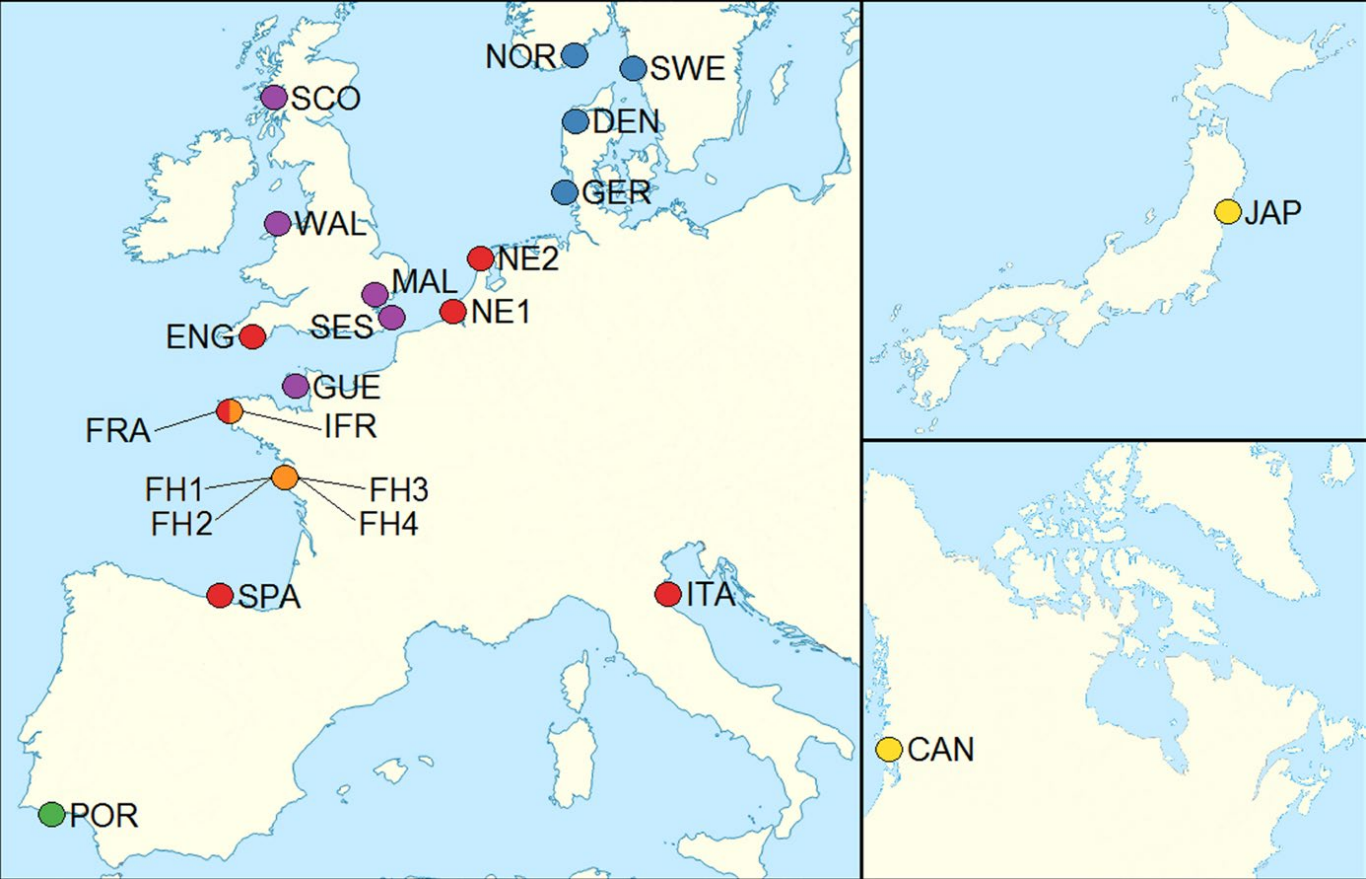
Map showing Pacific oyster sampling locations. The two source populations from the Miyagi Prefecture in Japan and British Columbia in Canada are indicated with yellow circles. Wild and hatchery populations within the southern group are indicated in red and orange, respectively, while wild populations and hatcheries within the northern group are represented by blue and purple circles, respectively. Finally, the population from Portugal, where hybridization between *C. gigas *and *C. angulata *is known to occur, is indicated by a green circle

Pacific oyster samples were collected between November 2014 and March 2016 from 12 different sites along the Atlantic seaboard of mainland Europe as well as from one location in the Mediterranean (Table 1 and Figure 1). Samples from Scotland (SCO) and Wales (WAL) were from hatcheries, while the remaining 11 populations were wild. Specimens from Portugal (POR) originated from an area where hybridization between *C. angulata *and *C. gigas* is known to take place (Batista et al., 2017; Huvet et al., 2004) and could therefore represent *C. gigas* samples introgressed with *C. angulata*. For comparison, we also included samples from the Miyagi Prefecture in Japan (JAP) and from British Columbia in Canada (CAN).

**Table 1:** Table of sampling locations including coordinates, origin classified as either wild or farmed, and the number of samples that were retained for analysis after quality control.

Population ID	Location	Latitude	Longitude	Origin	Sample size passing QC
POR	Faro, Portugal	37.002	−7.583	Wild	9
ITA	Ravenna, Italy	44.402	12.122	Wild	10
SPA	Santoña, Spain	43.426	−3.543	Wild	8
FRA	Brest, France	48.215	−4.462	Wild	10
ENG	Plymouth, UK	50.373	−3.441	Wild	9
NE1	Oosterschelde, Netherlands	51.608	3.91	Wild	12
NE2	Texel, Netherlands	53.001	4.474	Wild	10
IFR	Ifremer, France	48.351	−4.551	Hatchery	12
FH1	Hatchery 1, France	na	na	Hatchery	10
FH2	Hatchery 2, France	na	na	Hatchery	10
FH3	Hatchery 3, France	na	na	Hatchery	10
FH4	Hatchery 4, France	na	na	Hatchery	10
GUE	Guernsey, UK	49.497	−2.502	Hatchery	10
SCO	Oban, UK	55.534	−5.244	Hatchery	8
SES	Sea Salter, UK	51.378	1.212	Hatchery	9
MAL	Maldon, UK	51.724	0.71	Hatchery	9
WAL	Bangor, UK	53.098	−4.15	Hatchery	6
GER	Sylt, Germany	55.152	8.253	Wild	12
DEN	Limfjorden, Denmark	56.833	8.906	Wild	11
SWE	Kristineberg, Sweden	58.134	58.134	Wild	12
NOR	Arendal, Norway	58.428	8.793	Wild	11
JAP	Matsushima, Japan	38.367	141.066	Wild	12
CAN	Vancouver, Canada	50.164	−124.432	Wild	12
All	‐	‐	‐	‐	232

### 2.2 DNA extraction and SNP genotyping

Adductor muscle tissue was taken from each adult oyster and stored in 95% ethanol at −20°C. Whole genomic DNA was then extracted following an adapted phenol–chloroform protocol (Sambrook, Fritsch, & Maniatis, 1989) and sent to Edinburgh Genomics for genotyping at 40,625 SNPs on the custom Affymetrix SNP Array (Gutierrez et al., 2017). Out of a total of 204 DNA extracts, 192 (94%) passed quality checks and were therefore selected for genotyping using the protocol described by Gutierrez et al. (2017).

### 2.3 Incorporation of existing data

We also incorporated data into our study from 81 oysters that were previously genotyped on the same array (Gutierrez et al., 2017). These samples were initially included in the discovery panel used to develop and validate the SNP array and originated from eight hatcheries, three from the UK (MAL, SES, and GUE) and five from France (FH1–4 and IFR, Table 1 and Figure 1). In general, most companies use breeding practices based on rotational crosses among yearly cohorts (P. Harray, personal communication, November 21, 2018). Consequently, the genetic variability of a given cohort may not be representative of the population as a whole.

After the inclusion of these additional samples, our dataset consisted of (a) six wild populations from the southern group (red circles in Figure 1); (b) five hatcheries from the southern group (orange circles in Figure 1); (c) four wild populations from the northern group (blue circles in Figure 1); (d) five hatcheries from the northern group (purple circles in Figure 1); (e) the source populations of Japan and Canada (yellow circles in Figure 1); and (f) a single population from Portugal, where hybridization between *C. angulata *and *C. gigas* is known to occur (green circle in Figure 1).

### 2.4 SNP calling

We imported raw output data for all 273 samples into the Axiom Analysis Suite (version 3.1, Affymetrix) for quality control and genotype calling. All thresholds for quality assessment were set to the values recommended in the Affymetrix best practice workflow (Supporting information Table S1) and allowed for the categorization of each SNP into one of six possible classes: (a) “polymorphic high resolution” where the SNP passed all quality controls; (b) “no minor homozygote” where the SNP passed quality checks but no homozygotes for the minor allele were found; (c) “off‐target variant” where, in addition to the heterozygote and the two alternative homozygotes, a fourth genotype cluster was also observed; (d) “monomorphic high resolution” where the SNP passed quality checks but was uninformative; (e) “call rate below threshold” where the genotype call rate was below the specified threshold of 97%; and (f) “other” where the SNP failed to pass any other quality threshold. Following Affymetrix recommendations, SNPs from the first two categories were retained for further analysis, in addition to a subset of SNPs from the third category that were selected after applying the “off‐target caller” tool that allows for off‐target variant recalibration. The resulting dataset was then filtered to retain only SNPs genotyped in at least 90% of individuals and only samples with less than 5% missing data. Finally, the software PLINK (version 1.9, Purcell et al., 2007) was used to prune out linked loci using an *r* ^2 ^threshold of 0.5. The final dataset therefore comprised 232 individuals genotyped at 21,499 polymorphic, unlinked SNPs.

### 2.5 **RAD sequencing**

2.5

To provide a comparison with the SNP array data, we also RAD sequenced a representative subset of 40 individuals from eight populations (Supporting information Table S2). Specifically, we included the source population of Japan (JAP), the potential hybrid population from Portugal (POR), two geographically distant wild populations from the southern group (SPA and NE2), two wild populations from the northern group (DEN and NOR), the Mediterranean population from Italy (ITA), and a hatchery from Scotland (SCO). Only one hatchery could be included because DNA from the other hatcheries was either not of high enough quality to pass thresholds for library construction, or it was not available due to the sample having been genotyped as part of a previous study (Gutierrez et al., 2017). Whole genomic DNA was shipped to the Beijing Genomics Institute (BGI) for library preparation and sequencing. The libraries were constructed using the restriction enzyme PstI and sequenced on an Illumina X Ten platform to generate a total of 869,113,776 100 bp paired‐end sequence reads. Already demultiplexed sequence data were received from BGI and further sequence quality assessment was performed using the software FastQC (http://www.bioinformatics.babraham.ac.uk/projects/fastqc/). We then conducted a de novo assembly of the data and called genotypes using the Stacks 2.1 pipeline (Catchen, Hohenlohe, Bassham, Amores, & Cresko, 2013). Values for the three main parameters –*m*, –*M*, and –*n* were chosen following the optimization procedure described by Rochette and Catchen (2017). Briefly, –*m* was set to three, and increasing values for –*M* and –*n* were tested. The combination of these parameters for which the number of polymorphic loci present in at least 80% of the individuals reached a plateau was defined as optimal. Two different strategies were employed: –*n* was either set as equal to –*M* or one unit greater, to account for the potential presence of fixed *C. angulata* polymorphisms (Paris, Stevens, & Catchen, 2017). The combination yielding the highest plateau (*m* = 3, *M* = 5, and *n* = 6; Supporting information Figure S1) was selected for analyzing the entire dataset, from which PCR duplicates were then removed. The raw genotypes were subsequently quality filtered to retain only biallelic SNPs with both genotype quality and depth of coverage greater than 10 using VCFTools (Danecek et al., 2011). Subsequently, all SNPs and individuals with genotyping rates below 10% were removed and only variants with minor allele frequency (MAF) greater than 0.05 were retained. Finally, the software PLINK was employed to prune out linked loci using an *r* ^2 ^threshold of 0.2.

### 2.6 Analysis of population genetic structure

Three complimentary approaches were used to characterize the strength and pattern of population genetic structure. First, we calculated pairwise *F*_st _values among populations and determined their statistical significance based on 1,000 permutations of the dataset using Arlequin version 3.5.2.2 (Excoffier & Lischer, 2010). We then performed an analysis of molecular variance (AMOVA) employing the R package “poppr” version 2.8.0 (Kamvar, Brooks, & Grünwald, 2015; Kamvar, Tabima, & Grünwald, 2014) to evaluate the proportion of genomic variation explained by different hierarchical levels of population structure. Specifically, we started by dividing our data into four regions corresponding to the northern group, southern group, Portugal, and source populations. Each was then subdivided into wild and hatchery samples, which were further split based on the population of origin. Variation among samples within each population and within individuals was also evaluated. A randomization test with 1,000 repetitions was used to determine statistical significance. Subsequently, we used Mantel tests to evaluate the significance of isolation‐by‐distance patterns for the full dataset as well as for the wild and hatchery oysters separately for the northern and southern groups. For this analysis, geographic distances between populations were calculated as shortest coastline distances using “FreeMapTools (https://www.freemaptools.com/measure-distance.htm).” In the case of locations not connected by land, the shortest sailing distance between coasts was used. Second, we used the R package Adegenet version 2.1.1 (Jombart, 2008; Jombart & Ahmed, 2011) to conduct a principal component analysis (PCA) of the SNP dataset. Third, we utilized the software package fineRADstructure (Malinsky, Trucchi, Lawson, & Falush, 2018) to infer population structure using a model‐based Bayesian clustering approach that groups together individuals with high levels of shared coancestry. A “coancestry matrix,” defined as a summary of nearest neighbor haplotype relationships, is required as input and was generated using the “RADpainter” module of fineRADstructure. We used the default parameters of 100,000 Markov chain Monte Carlo (MCMC) iterations with a burn‐in of 100,000 iterations and sampling occurring every 1,000 iterations. A tree was then constructed with 10,000 hill‐climbing iterations, and the results were visualized using the scripts FINERADSTRUCTUREPLOT.R and FINESTRUCTURELIBRARY.R, which are available via http://cichlid.gurdon.cam.ac.uk/fineRADstructure.html.

### 2.7 Genomic inbreeding coefficients

We calculated ${\hat{F}}_{I}$, a genomic inbreeding estimator based on the variance of additive genotype values (Yang, Lee, Goddard, & Visscher, 2011), for each individual in our dataset based on the SNP data. To test for an association between levels of relatedness and inbreeding, we calculated mean pairwise relatedness among individuals within populations from the SNP data using GCTA (Yang et al., 2011) and correlated this with mean ${\hat{F}}_{I}$ values. Genomic inbreeding coefficients were compared between populations and groups using a Kruskal–Wallis test followed by *post hoc* pairwise Mann–Whitney tests, whose *p*‐values were adjusted according to Benjamini and Hochberg (1995), to formally test for significant pairwise comparisons. As variation in inbreeding causes heterozygosity to be correlated across loci, we also estimated the extent of identity disequilibrium (ID, Weir & Cockerham, 1973) by calculating the two‐locus heterozygosity disequilibrium, *g_2_* (David, Pujol, Viard, Castella, & Goudet, 2007) within the R package inbreedR (Stoffel et al, 2016). The same package was also used to calculate the 95% confidence interval of *g_2_* by bootstrapping the data 1,000 times over individuals, as described by Stoffel et al. (2016).

## 3 RESULTS

To provide detailed insights into the pan‐European population structure of *C. gigas* and facilitate comparisons between wild and hatchery oysters, we analyzed medium‐density SNP array data for a total of 273 individuals sampled from 23 locations. Data from 192 individuals were newly generated, while the remaining data were incorporated from Gutierrez et al. (2017). Sampling sites were putatively assigned to either the northern or the southern group on the basis of previous genetic studies (Lallias et al, 2015; Rohfritsch et al, 2013). Application of the filtering criteria described in the Materials and methods resulted in the exclusion of an 17,411 SNPs that did not meet Affymetrix recommendations and of an additional 1,715 SNPs due to low genotyping rates or linkage disequilibrium. On average, 10 individuals were genotyped for each location, and the final dataset consisted of 232 samples (see Table 1 for a breakdown by population) genotyped at 21,499 SNPs.

### 3.1 Population genetic structure

To investigate broad‐scale patterns of genetic differentiation, we used AMOVA to quantify the proportion of genomic variation attributable to each of five hierarchical levels of population substructure. As expected, over 95% of the total variation was partitioned within individuals. The remaining variance was mainly partitioned among the northern and southern groups, the source populations, and Portugal (Φ = 0.017, *p* = 0.024, Table 2), between wild populations and hatcheries (Φ = 0.004, *p* = 0.005, Table 2) and among sampling locations (Φ = 0.02, *p* = 0.001, Table 2). Furthermore, the majority of pairwise *F*_st_ values between populations were highly significant, even after correction for multiple tests (Supporting information Table S3), although a significant isolation‐by‐distance pattern was only detected among wild populations belonging to the southern group (Mantel's *r* = 0.971; *p* = 0.022).

**Table 2:** Results of the hierarchical analysis of molecular variance (AMOVA)

Source of variation	*df*	Sum of squares	% variation	*Φ*	*p*‐value
Among regions	3	48,727.66	1.7	0.017	0.024
Between origins within regions	2	17,734.93	0.39	0.004	0.005
Among sampling locations within origins	17	1,16,998.80	2	0.02	0.001
Among samples within sampling locations	209	10,14,805.34	0.22	0.002	0.406
Within samples	232	11,21,225.26	95.69	0.043	0.001
Total	463	23,19,491.99	100	‐	‐

Notes

Five different hierarchical levels were evaluated. First, the dataset was divided into four “regions” corresponding to the southern group, the northern group, the source populations, and Portugal. Next, each region was divided into “origins” depending whether the samples were from wild populations or hatcheries. Finally, the remaining variance was partitioned among sampling locations, individuals within sampling locations, and within individuals.

**Figure 2:**
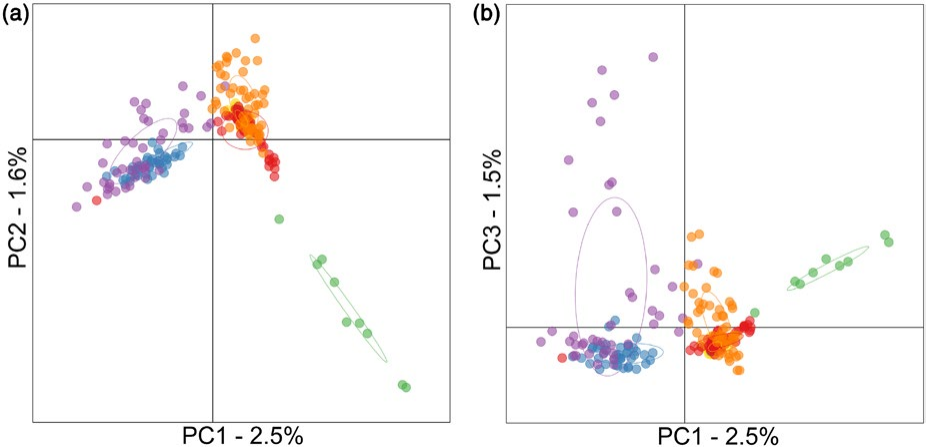
Scatterplot showing individual variation in principal component (PC) scores derived from a principal component analysis (PCA) of the genomic data. Panel (a) shows PC1 plotted against PC2, and panel (b) shows PC1 plotted against PC3. The amounts of variation explained by each PC are given as percentages. Samples are color coded as described in the legend of Figure 1

To evaluate population structure at the individual level, we performed a principal component analysis (PCA). Consistent with the AMOVA, a number of clear differences were apparent. First, the northern and southern groups clearly separated apart from one another, as did oysters from Portugal, although no genetic differences were apparent between the southern group and the two source populations (Figure 2). Furthermore, within both the northern and southern groups, hatcheries showed consistently greater scatter than wild populations, indicating that they may have experienced stronger genetic drift.

**Figure 3:**
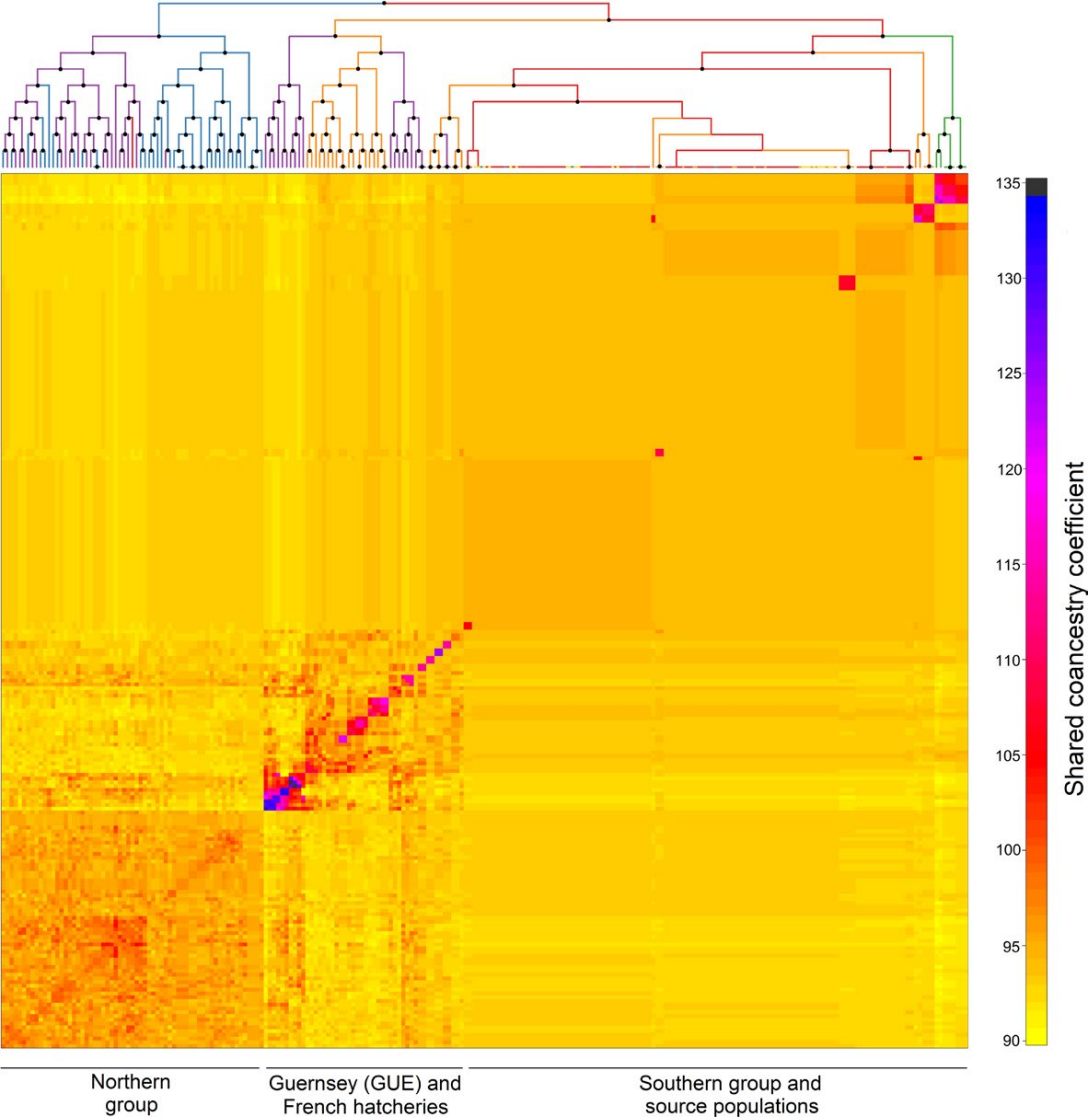
Output of the fineRADstructure analysis of the genomic data. In the cladogram, branches are color coded according to sampling origin as shown in Figure 1 and nodes with greater than 95% bootstrap support are marked by black points. The heatmap indicates pairwise coancestry between individuals, with blue and purple representing the highest levels, red and orange indicating intermediate levels, and yellow representing the lowest levels of shared coancestry

We next used a model‐based Bayesian clustering approach implemented in fineRADstructure to infer population structure via shared ancestry. The resulting cladogram and coancestry matrix shown in Figure 3 confirmed the results of the AMOVA and PCA while also uncovering the presence of more subtle structuring. Specifically, two major clades were identified. The first of these, shown on the left of the cladogram and represented by a cluster of individuals in the bottom left of the coancestry matrix, comprised individuals from the northern group. This was further subdivided into two distinct clusters, the first comprising mainly individuals from the Seasalter, Maldon, and Bangor hatcheries in the UK and the second comprising mostly wild individuals from Germany and Scandinavia. The remaining individuals were grouped together into a second major clade shown on the right of the cladogram, which in turn was subdivided into three main clusters comprising the southern group and source populations, oysters from the Guernsey, Seasalter, and two of the French hatcheries, and Portugal. The fact that samples from Seasalter were distributed across two different clusters is consistent with the fact that oysters have been exchanged between Seasalter and Guernsey (M. Dravers, personal communication, November 21, 2018). Most of the individuals from the remaining French hatcheries could also be clearly distinguished within the southern group. Samples from Scotland were in a different part of the cladogram, but always clustered together with other samples from hatcheries. Furthermore, levels of shared coancestry varied appreciably across the dataset, with oysters from the Guernsey hatchery having the highest levels, the remaining hatchery samples as well as oysters from Portugal having intermediate levels, and wild individuals having the lowest levels.

### 3.2 Variation in inbreeding

**Figure 4:**
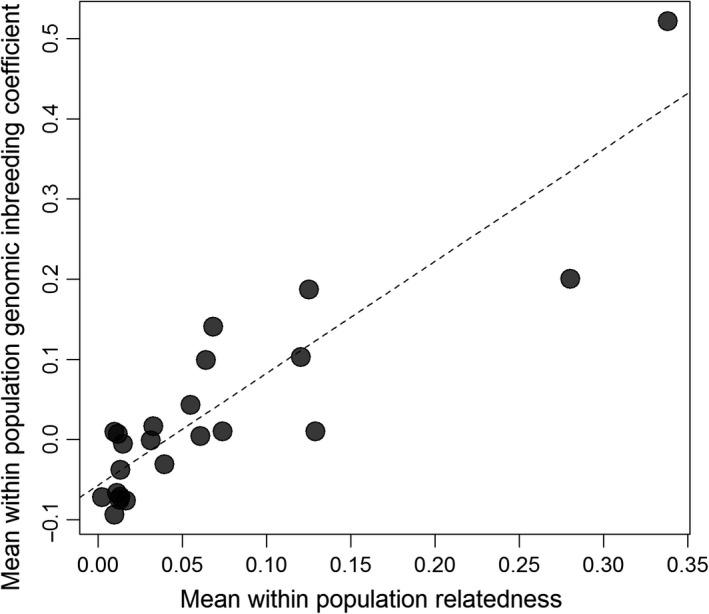
Relationship between average pairwise relatedness and average genomic inbreeding coefficient within populations (linear regression, *b = *1.39, *r* ^2^ = 0.79, *p* < 0.001)

To explore patterns of inbreeding in wild populations and hatcheries, we calculated genomic inbreeding coefficients for all of the individuals in our dataset. Mean genomic inbreeding coefficients were strongly positively correlated with average pairwise relatedness values within populations (linear regression, *b = *1.39, *r* ^2^ = 0.79, *p* < 0.001, Figure 4), which in turn were tightly correlated with mean shared coancestry values extracted from the fineRADstructure analysis (linear regression, *b = *48.99, *r* ^2^ = 0.99, *p* < 0.001). This indicates that, as expected, genomic inbreeding coefficients tend to be higher in populations with elevated levels of relatedness and shared coancestry.

**Figure 5:**
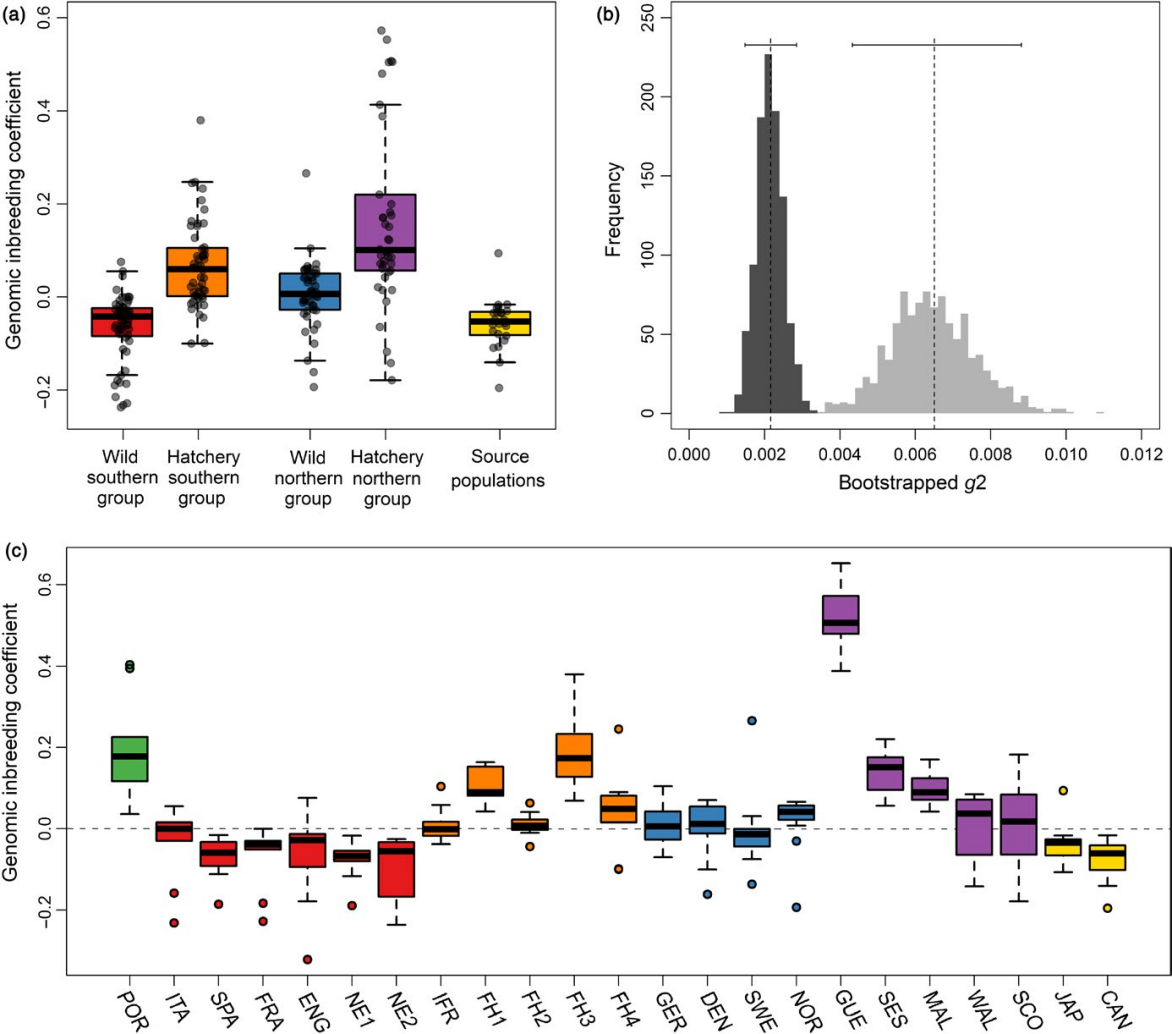
Levels of inbreeding in wild populations and hatcheries inferred from genome‐wide SNP data. Panel (a) shows differences between wild and hatchery samples from the northern and southern groups separately as well as for the source populations. Raw data points are shown together with standard Tukey box plots. Panel (b) shows bootstrapped g_2_ values for individuals sampled from wild populations (dark gray) versus hatcheries (light gray). The empirical g_2_ values and their corresponding 95% confidence intervals are depicted by dashed vertical lines and horizontal bars, respectively. Panel (c) shows population‐specific variation in inbreeding. In panels (a) and (c), the populations are color coded as described in the legend of Figure 1

Pooling individuals according to the six main groups described in the first paragraph of the results revealed highly significant differences (Figure 5a, Kruskal–Wallis test: χ^2^ = 105.17, *df* = 4, *p* < 0.001). First of all, regardless of whether wild populations or hatcheries were considered, a significant tendency was found for genomic inbreeding coefficients to be higher in the northern than in the southern group (*post hoc* pairwise Mann–Whitney tests: wild: adjusted *p < *0.001, hatchery: adjusted *p = *0.005). This is consistent with previous studies showing that genetic diversity is lower in the northern group (see Discussion). Second, genomic inbreeding coefficients were significantly higher in hatcheries versus wild populations (*post hoc* pairwise Mann–Whitney tests: northern group: adjusted *p* < 0.001, southern group: adjusted *p* < 0.001). In line with this, identity disequilibrium was higher in individuals sampled from hatchery (*g*_2_ = 0.0065, bootstrap 95% confidence interval = 0.0044–0.0089) than from wild populations (*g*_2_ = 0.0022, bootstrap 95% confidence interval = 0.0014–0.0029), indicating that hatchery‐reared oysters have greater overall variance in inbreeding (Figure 5b). This reflects a general tendency for the variance in inbreeding to be higher both within and among hatcheries relative to wild populations (Figure 5c). The highest genomic inbreeding coefficients were found in oysters from Guernsey, while in France and England, hatcheries with both intermediate (e.g., Fh1, FH3, SES, and MAL) and relatively low (e.g., IFR, FH2, FH4, WAL, and SCO) levels of inbreeding were present.

### 3.3 RAD sequencing

**Figure 6:**
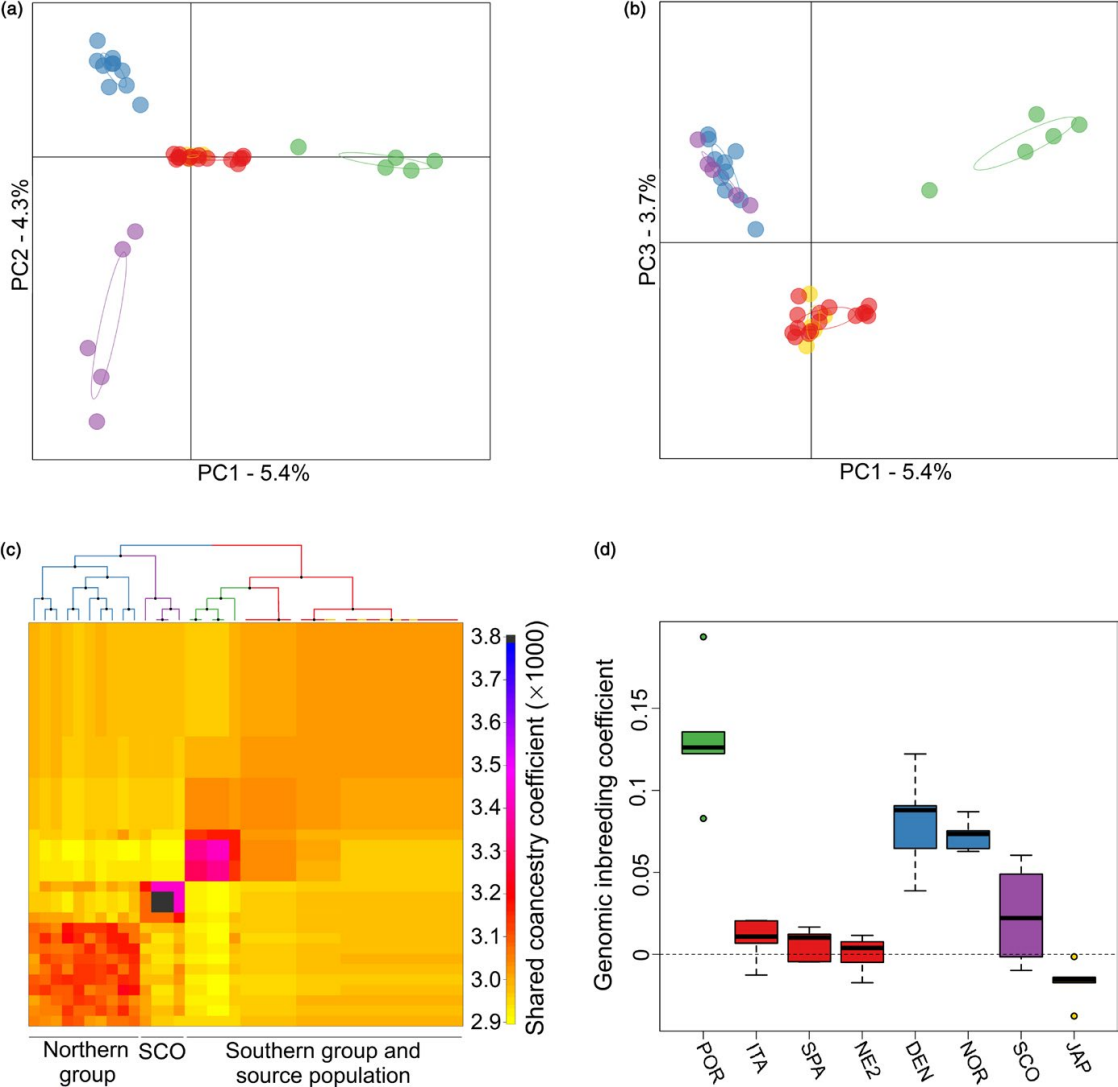
Results of repeated analyses of population genetic structure and inbreeding based on a subset of RAD sequenced individuals from eight populations (see Methods for details). Panels (a) and (b) show individual variation in principal component (PC) scores derived from a principal component analysis (PCA). Panel (a) shows PC1 plotted against PC2, while panel (b) shows PC1 plotted against PC3. The amounts of variation explained by each PC are given as percentages. Panel (c) presents the output of the fineRADstructure analysis, including both the cladogram and the heatmap representing pairwise coancestry values between individuals. Panel (d) shows population‐specific variation in inbreeding. Samples are color coded as described in the legend of Figure 1

Although we did not expect our results to be strongly affected by ascertainment bias because oysters from both the northern and southern groups were used in the discovery panel for the SNP array (Gutierrez et al., 2017), we nevertheless generated for comparison a parallel genetic dataset comprising RAD sequencing data for 40 individuals from eight populations (see Materials and methods for details). This resulted in a total of 869,113,776 high‐quality paired‐end reads that were assembled into 697,354 RAD loci. From these, we called a total of 7,322,935 SNPs, of which 115,087 were retained for further analyses after filtering. We found a virtually identical pattern of population structure, with the PCA clearly discriminating the northern group from the southern group, the wild populations from the hatcheries, and oysters from Portugal (Figure 6a and b). Similarly, fineRADstructure identified four main groups comprising the southern group and source population, the northern group, Portugal, and the Scottish hatchery (Figure 6c). Genomic inbreeding coefficients based on the RAD data showed a similar pattern to those calculated from the SNP array (Figure 6d), although inbreeding levels appeared to be somewhat higher for the Danish and Norwegian populations. Notably, oysters from Portugal had the highest levels of inbreeding in our RAD analysis, which would not be expected if our sample contained hybrids. Nevertheless, due to the high pairwise *F*_ST_ values obtained in all comparisons involving oysters from Portugal (Supporting information Table S3), we cannot exclude the possibility that these samples may actually be pure *C. angulata* rather than pure *C. gigas. (see Discussion)*

## 4 DISCUSSION

We used a medium‐density SNP array to characterize the genetic structure of *C. gigas* populations across Europe as well as to evaluate levels of inbreeding in wild and hatchery oysters. Our comprehensive sampling design coupled with high‐resolution genomic data allowed us to resolve patterns of genetic differentiation over both broad and fine geographic scales. Specifically, we found clear support for a northern and southern European group, with the latter being virtually identical to the Japanese and Canadian source populations, consistently with previous studies (Huvet, Lapegue, Magoulas, & Boudry, 2000; Moehler et al., 2011; Rohfritsch et al., 2013). We furthermore resolved substantial genetic differences between wild populations and hatcheries and compared genomic inbreeding coefficients to show that some of the sampled hatchery cohorts have higher levels of inbreeding than wild populations. Given that *C. gigas *carries a high genetic load that has been proposed to be responsible for substantial early mortality (Launey & Hedgecock, 2001; Plough & Hedgecock, 2011; Taris et al., 2007) as well as variation in commercially important adult traits (Evans et al., 2004), we believe that our findings could have important implications for aquaculture.

### 4.1 Population genetic structure

Several studies have investigated the population genetic structure of Pacific oysters in Europe and interpreted their findings in the light of the known and rather complex history of multiple introductions and invasions. Our research compliments and builds upon these studies in a number of ways. First, we were able to confirm previous findings based on mitochondrial DNA as well as small panels of nuclear markers (Huvet et al., 2000; Lallias et al., 2015; Meistertzheim et al., 2013; Moehler et al., 2011; Rohfritsch et al., 2013) that European Pacific oyster populations are broadly structured into northern and southern groups. Although this is not necessarily surprising, studies based on one or a few markers can suffer from biases related to stochastic processes (Rokas & Carroll, 2005). Consequently, our study lends further weight to the conclusion that the north–south divide is a genome‐wide phenomenon that is robust to different methodologies and repeatable across studies.

We were also able to confirm previous studies (Huvet et al., 2000; Moehler et al., 2011; Rohfritsch et al., 2013) reporting negligible genetic differentiation between the source population of Japan and the southern European group. Despite having analyzed samples from both Japan and British Columbia, which was a secondary site of introduction into Europe (Wolff & Reise, 2002), and having several orders of magnitude higher genetic resolution than previous studies, both PCA and fineRADstructure failed to detect any clear differences between the southern group and source populations. Furthermore, although a number of comparisons involving Japan and British Columbia yielded significant *F*_st_ values, the magnitude of these estimates was low. Our results therefore lend additional weight to the notion that Pacific oysters did not experience a pronounced founder effect when they were introduced into southern Europe. This is consistent with the observation that many thousands tonnes of spat were transferred into northern France from Japan as well as many hundreds of tonnes of adults from British Columbia (Grizel & Héral, 1991; Nehring, 1999; Wolff & Reise, 2002).

In addition to confirming previous findings, our genomic data also allowed us to resolve fine‐scale patterns that could not be detected in previous studies. In particular, Huvet et al. (2004), Meistertzheim et al. (2013), and Rohfritsch et al. (2013) did not find any significant genetic differences among populations of the southern group, regardless of whether mitochondrial or nuclear markers were used. This apparent homogeneity was attributed to the prodigious reproductive potential of this species coupled with the possession of long‐lived pelagic larvae and frequent transfers of farmed stocks (Meistertzheim et al., 2013; Rohfritsch et al., 2013). By contrast, we not only found that a number of pairwise population comparisons within the southern group yielded significant* F*_st_ values (Supporting information Table S3) but also uncovered a significant isolation‐by‐distance pattern among the wild populations from southern Europe. The highest *F*_st_ values in southern Europe were obtained for comparisons involving Italy, presumably due to the fact that *C. gigas* was introduced into this part of the Mediterranean during the late 1960 s (Šegvić‐Bubić et al., 2016). We also found some evidence for the presence of fine‐scale population structure within the northern cluster, although this was more equivocal. Specifically, most but not all of the individuals from Scandinavia and Germany clustered apart from the British hatcheries in the fineRADstructure analysis, although this distinction was not readily apparent in the PCA. Such a pattern is consistent with Pacific oysters having been imported repeatedly from UK hatcheries to Germany and Scandinavia (d'Auriac et al., 2017).

### 4.2 Comparison of wild populations and hatcheries

Two innovations of our study were first to sample wild and hatchery oysters extensively enough to facilitate a meaningful and broad‐scale comparison, and second to quantify inbreeding directly from genomic data. Repeated introductions of genetic material from different aquaculture broodstocks are commonplace and should in principle contribute toward the genetic homogenization of wild populations and hatcheries (Moehler et al., 2011). Moreover, a certain degree of genetic exchange between wild populations and hatcheries can be expected, at least in France where oyster production in some hatcheries is partially based on wild‐caught spat and natural reproduction of farmed oysters occurs (Pouvreau et al., 2016). Set against this, however, temporal sweepstake effects (Hedgecock & Pudovkin, 2011) and far smaller numbers of breeding individuals in aquaculture populations (Kochmann et al., 2012) could potentially increase genetic drift and drive genetic differentiation from wild populations. Our data lend support to the latter scenario as we found that hatcheries showed a clear tendency to cluster apart from wild populations and were also characterized by elevated levels of shared coancestry and inbreeding.

Although small panels of genetic markers like microsatellites are capable of resolving population structure, under most circumstances they provide poor estimates of inbreeding (Balloux et al, 2004). This has hindered the study of inbreeding in wild populations lacking pedigrees (Pemberton, 2008). Consequently, we used our SNP data to calculate genomic inbreeding coefficients for the first time to our knowledge for a marine invertebrate in order to investigate how aquaculture practices may have influenced levels of inbreeding in oyster hatcheries. We uncovered a clear tendency for both the magnitude of inbreeding and its variance to be higher within the sampled hatchery cohorts. This might be considered surprising given that *C. gigas* is produced in vast numbers and is capable of long‐distance dispersal mediated by free‐swimming planktotrophic larvae (FAO, http://www.fao.org/fishery/culturedspecies/Crassostrea_gigas). However, Pacific oysters also have one of the smallest documented effective to census population size ratios (10^−^^6^, Frankham, 1995) reflecting a general tendency in marine invertebrates for highly variable sweepstakes reproductive success resulting from a combination of high fecundity and low larval survivorship (Hedgecock & Pudovkin, 2011). Concretely, a single oyster can produce several tens of millions eggs in a single season (FAO, http://www.fao.org/fishery/culturedspecies/Crassostrea_gigas), but mortality rates within commercial oyster hatchery cultures can be as high as 98% between fertilization and the spat stage (Plough & Hedgecock, 2011), which may lead to high variance in the reproductive success of hatchery broodstock (Boudry, Collet, Cornette, Hervouet, & Bonhomme, 2002).

Our findings are in line with a previous study documenting lower microsatellite allelic diversity in hatchery‐reared relative to wild individuals within Loch Foyle in Northern Ireland (Kochmann et al., 2012), although a similar study did not find any differences between wild populations and hatcheries in the Wadden Sea (Moehler et al, 2011). Moreover, heterozygote deficiency has been observed in several previous studies of oysters (Lallias et al., 2015, 2010; Meistertzheim et al., 2013; Rohfritsch et al., 2013), which has been interpreted as being suggestive of inbreeding (Faust et al., 2017). Finally, experimental studies have observed massive distortions in marker segregation ratios in *F*_2_ oyster families, consistent with a high genetic load comprising multiple recessive mutations under strong viability selection (Launey & Hedgecock, 2001; Plough & Hedgecock, 2011). Hence, our study contributes toward a growing body of evidence in support of Launey and Hedgecock's (2001) argument that inbreeding may be a biologically and economically important phenomenon in oysters as well as possibly in other marine invertebrates.

It is important to recognize that not all of the hatchery‐reared oysters in our study showed higher levels of inbreeding than wild populations. By implication, inbreeding is not associated with hatchery propagation *per se* but may rather arise due to differences in management practices among facilities, which in many cases will reflect differing priorities. For example, many hatcheries minimize the risk of inbreeding by enhancing their broodstock with oysters collected from the wild (E. Vernier, *personal communication*, November 21, 2018) or by maximizing numbers of effective breeders, while others actively avoid these practices (M. Montergous, *personal communication*, June 6, 2018), presumably to minimize the risk of disease transmission. In other cases, it may be desirable to maintain particular families or lineages that have been selected based on specific characteristics, even if this results in somewhat higher levels of consanguinity (M. Dravers, *personal communication*, September 6, 2018).

### 4.3 Practical implications for oyster aquaculture

Moderate to high levels of inbreeding are known to negatively impact a multitude of commercially relevant fitness traits, from individual growth rate through harvest body size to survival, in many aquaculture organisms (Deng et al., 2005; Gallardo et al., 2004; Lyu et al., 2018; Moss et al., 2007). More specifically, previous studies of oysters have found strong inbreeding depression for early viability (Plough & Hedgecock, 2011) as well as for yield, growth rate, and survival to harvest in adults (Evans et al., 2004). Consequently, elevated inbreeding levels in certain hatcheries are worthy of further exploration and it may be worth considering intervention strategies aimed at increasing genetic diversity.

With respect to the need for further exploration, it is worth bearing in mind that although our total sample size of oysters was reasonably large given the number of markers deployed, only around ten samples were analyzed on average from each population. While this is unlikely to have appreciably affected our inference of population structure (Willing, Dreyer, & Oosterhout, 2012), the inference of inbreeding levels within populations could be sensitive to the inadvertent sampling of highly related individuals when sample sizes are small, and this may be particularly true for hatcheries. Furthermore, high variance in reproductive success within and across generations could potentially lead to different cohorts from the same hatcheries varying substantially in their levels of inbreeding. Consequently, it would be worthwhile enlarging sample sizes within hatcheries, as well as collecting and analyzing samples from the same locations over multiple years in order to provide more robust inferences of average inbreeding levels and allow these to be interpreted in the light of temporal variation. This would further benefit from the development of a larger SNP array and a more contiguous *C. gigas* reference genome, which would allow inbreeding to be evaluated with even greater precision through the use of mapped genetic markers to quantify runs of homozygosity.

Having done so, a useful next step would be to evaluate in greater detail the effects of different levels of inbreeding on commercially important traits within hatcheries. So far, only a single study has evaluated the effects of inbreeding on adult traits in a commercial growing environment and this was based on a crossing design that maximized variation in inbreeding among families (Evans et al., 2004). By contrast, our approach of quantifying inbreeding directly from genomic data could in principle circumvent the need for experimental crosses, thereby allowing inbreeding depression to be directly quantified in real hatchery populations when phenotypic data are available. This could help to inform hatchery managers about the potential costs of inbreeding and the possible benefits of intervention strategies.

Finally, a number of potential intervention strategies aimed at reducing inbreeding and increasing genetic diversity could be envisaged. The first obvious approach would be to incorporate individuals from wild populations into hatchery broodstocks, as also discussed by Lallias et al. (2010) in the context of flat oysters. However, caution is warranted as selective breeding in captivity may lead to adaptive changes that are absent from wild populations (Lachambre et al., 2017) so the fitness consequences of such crosses remain unclear. A second possibility would be exchange individuals more extensively among hatcheries. Within Europe, the practice of exchanging oyster stocks between different countries is becoming more common, but we are not aware of any such exchanges between the United Kingdom and the European mainland, probably due to the perceived risk of disease transmission. A third possibility would be to mitigate the risk of inbreeding by implementing oyster rearing based on molecular pedigree assignments (Boudry, 2009; Lapegue et al., 2014) as is common practice in fish farming (Vandeputte & Haffray, 2014). Clearly, hatchery managers need to balance the pros and cons of selective breeding and maximizing genetic diversity, but either way genomic tools such as SNP arrays provide a means of evaluating the genetic consequences of chosen management practices.

### 4.4 Caveats

SNP arrays provide a cost‐effective and convenient route to genome‐wide investigations but can be prone to ascertainment bias when the samples used in the initial SNP discovery phase differ from those being interrogated on the array (Lachance & Tishkoff, 2013). However, we believe this is unlikely to substantially affect our main conclusions for two reasons. First, the discovery panel of individuals used to construct the array was unusually large, comprising over 200 individuals from eight different localities. By implication, much of the genetic diversity of the species in Europe should have been captured, including rare alleles that may easily be missed with smaller discovery panels but which could potentially be present at higher frequencies in unsampled populations. Second, although the discovery panel comprised primarily individuals from hatcheries, the northern and southern groups were roughly equally represented and we therefore have no reason to expect any broad‐scale biases to be present. Two further points should also be recognized. First, our analyses of population structure will if anything be conservative, as ascertainment bias should lead to the underestimation of genetic differentiation when peripheral populations carry previously undetected alleles. Second, ascertainment bias cannot explain higher levels of inbreeding nor variation in inbreeding among hatcheries in the UK and France. This is because all of these populations were used to generate the array, so ascertainment bias if present would be expected to generate the opposite pattern of increased homozygosity in wild populations.

Nevertheless, we conservatively took into account the possibility that ascertainment bias could be responsible for the ostensibly high level of inbreeding in the putatively hybrid Portuguese population. To test this possibility as well as to confirm our broader findings, we RAD sequenced a subset of individuals and repeated all of our analyses. Our previous results were largely confirmed, with very similar patterns of population genetic structure and inbreeding being obtained, lending further weight to our main conclusions. Furthermore, oysters from Portugal were again found to have relatively high genomic inbreeding coefficients based on the RAD data. As we would expect hybrids to be relatively outbred, this finding points toward hybridization between *C. gigas* and *C. angulata *being negligible in our sample. Consequently, it appears that the Portuguese oysters could represent and inbred and isolated *C. gigas* population. However, we cannot discount the further possibility that we inadvertently sampled *C. angulata* from this location, as the two species are morphologically indistinguishable, the SNP array may include loci that cross amplify in *C. angulata* (Gagnaire et al., 2018), and *F*_st _comparisons involving our Portuguese sample were consistently high.

## 5 CONCLUSION

We harnessed some of the latest developments in genomics to shed new light on the population structure of Pacific oysters along a European latitudinal cline as well as to compare levels of inbreeding between wild and hatchery samples. The several orders of magnitude higher genetic resolution provided by the medium‐density SNP array allowed us not only to confirm previous findings (Faust et al., 2017; Lallias et al., 2015; Meistertzheim et al., 2013; Rohfritsch et al., 2013) but also to detect fine‐scale patterns including genetic differences between wild populations and hatcheries. We furthermore uncovered evidence for higher levels of inbreeding in sampled hatchery cohorts, which merits further investigation. Finally, our study contributes to a growing consensus that inbreeding could be more prevalent in animal populations than previously envisaged (Keller & Waller, 2002), even in highly fecund species with high dispersal.

## CONFLICT OF INTEREST

None Declared.

## Data Availability

Individual genotype files obtained from the SNP array and from the analyses of the RAD sequencing data are available from the Dryad Digital Repository: https://doi.org/10.5061/dryad.6d778b6
