# Supplementary figures and images for: Detailed insights into pan‐European population structure and inbreeding in wild and hatchery Pacific oysters (Crassostrea gigas) revealed by genome‐wide SNP data
Source: Evol Appl. 2018 Dec 31;12(3):519–34. doi: 10.1111/eva.12736 (PMC6383735; doi:10.1111/eva.12736)

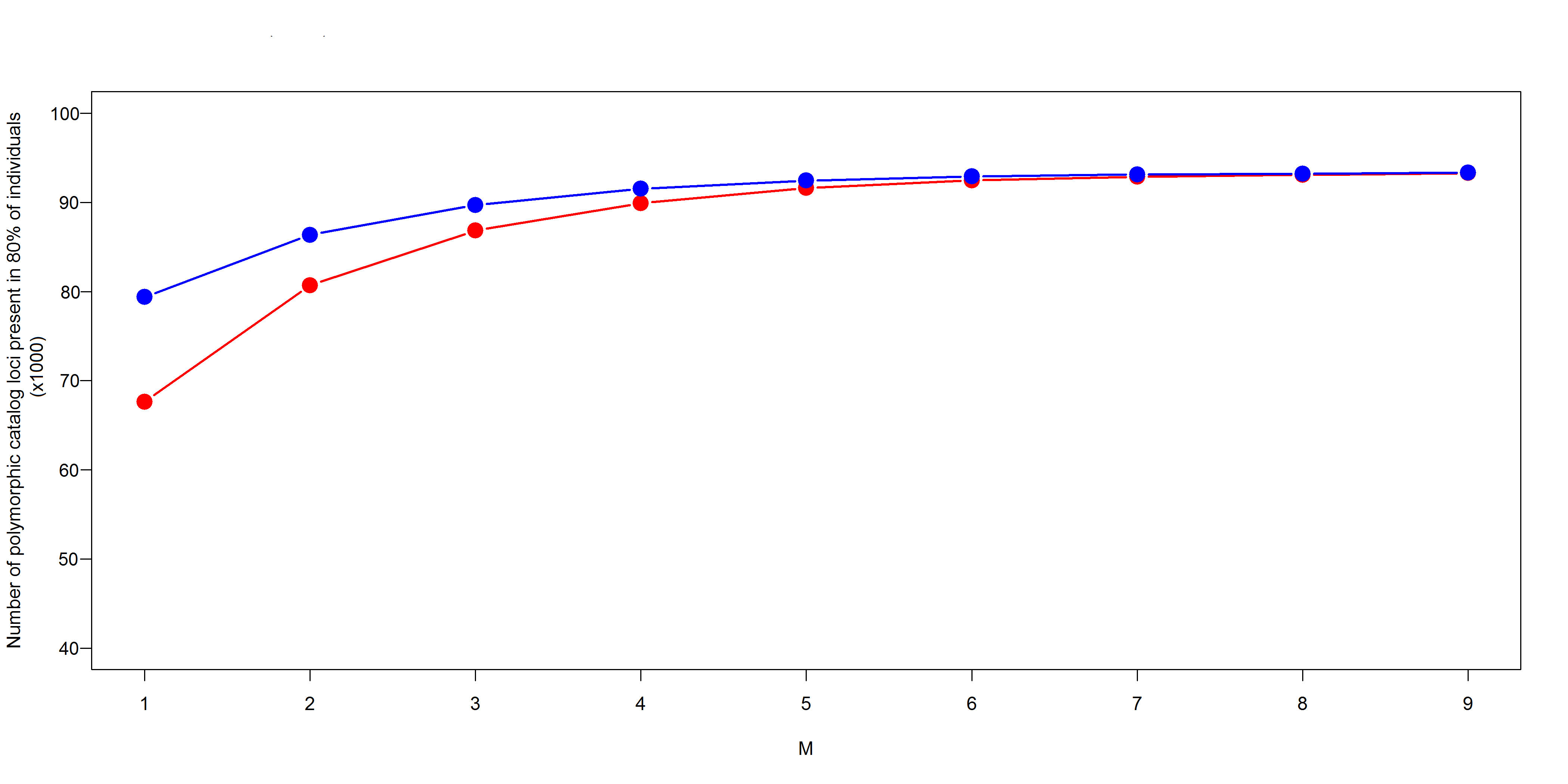

Supplement: Supplementary file 1 [file EVA-12-519-s001.tif]
